# Aortic emergencies—diagnosis and treatment: a pictorial review

**DOI:** 10.1007/s13244-014-0380-y

**Published:** 2015-02-01

**Authors:** Esther Voitle, Wolfgang Hofmann, Manfred Cejna

**Affiliations:** 1 Institute for Diagnostic and Interventional Radiology, Academic Teaching Hospital LKH Feldkirch, Carinagasse 47, 6800 Feldkirch, Austria; 2 Department of Vascular Surgery, Academic Teaching Hospital LKH Feldkirch, Carinagasse 47, 6800 Feldkirch, Austria

**Keywords:** Aortic pathology, Aortic Dissection, Aortic Aneurysm, Diagnostic imaging, Endovascular aortic repair (EVAR)

## Abstract

**Objectives:**

To demonstrate the various presentations of acute aortic pathology and to present diagnostic and therapeutic approaches.

**Methods:**

Diagnostic imaging is the key to the reliable diagnosis of acute aortic pathology with multi-slice computed tomography angiography (CTA) as the fastest and most robust modality. Endovascular aortic repair (EVAR) with stent grafts and open surgical repair are therapeutic approaches for aortic pathology.

**Results:**

CTA is reliable in diagnosing and grading aortic trauma, measuring aortic diameter in aortic aneurysms and detecting vascular wall pathology in acute aortic syndrome and aortic inflammation. CTA enables planning the optimal therapeutic approach. Stent graft implantation and/or an open surgical approach can address vascular wall pathology and exclude aortic aneurysms.

**Conclusion:**

Aortic emergencies have to be detected quickly. CTA is the imaging method of choice and helps to decide whether elective, urgent or emergent treatment is necessary with EVAR and open surgical repair as the main treatment approaches.

***Teaching Points*:**

• *To present aortic pathology caused by trauma*

• *To present acute aortic syndrome (aortic dissection, intramural haematoma and penetrating ulcers)*

• *To present symptomatic and ruptured aortic aneurysm*

• *To present infection (mycotic aneurysms/aorto-duodenal fistulae) or iatrogenic injury of the aorta*

• *To understand different presentations for treatment planning (EVAR and open surgery)*

## Introduction

Aortic emergencies present a diagnostic and treatment challenge for emergency physicians [1].

Aortic emergencies comprise different clinical entities with only one thing in common: if not diagnosed and managed in due time they are life threatening [2–5]. Compared to myocardial infarction and stroke, aortic pathologies are relatively rare (Table 1). A rapid diagnosis and treatment decision is therefore necessary to decrease mortality. CTA has become the best modality for the diagnosis and treatment planning of aortic pathologies [3, 4, 6, 7]. Treatment is performed by either an open surgical approach or endovascular stent graft implantation (endovascular aortic repair, EVAR) [3, 7–9].

**Table 1:** Incidences of various aortic pathologies compared to myocardial infarction and stroke

	Incidence
Myocardial infarction	80–330 patients/100,000 patients/year at the age of 45–54 and 350–1,140 patients/100,000 patients/year at the age of 65–74 [2]
Stroke	180–294 patients/100,000 patients/year [2]
Acute aortic syndrome	The majority of patients present with aortic dissection (AD); its incidence is estimated to be 2–3.5 patients/100,000 patients/year [3]. Accurate numbers are difficult to obtain because just like in the case of ruptured aneurysms many patients may die without proper diagnosis, without reaching a hospital and/or receiving treatment
Thoracic aortic aneurysm	The incidence of thoracic aortic aneurysms is approximately 10 cases per 100,000 person-years [4], estimated to be increasing
Abdominal aortic aneurysm	In the abdominal aorta aneurysms can be found in as many as 7 % of patients when screening in the >65-year population [5]. Studies showed the highest prevalence of AAA >3.0 cm was 5.9 % and was found in white male smokers between 50 and 79 years [6]
Symptomatic aneurysm (abdominal)	Symptomatic AAA in males has an incidence of 25 per 100,000 at age 50, increasing to 78 per 100,000 over the age of 70 [7]
Ruptured aneurysm (abdominal)	The incidence of ruptured abdominal aortic aneurysms ranges between 5.6 and 17.5 per 100,000 person-years in Western countries [7]. The incidence of AAA is estimated to be decreasing, as well as the incidence of AAA rupture [7]
Aorto-enteric fistulae (AEF)	AEFs are very rare with an incidence of 0.1/100,000 patients/year, mostly secondary aorto-enteric fistulae in patients with a history of aortic aneurysm and/or surgical aortic repair. Primary aorto-enteric fistulae without previous surgical aortic repair have an even lower incidence

## Computed tomography imaging

Currently, the most often used imaging modality in emergency radiology is computed tomography (CT) imaging [3, 6, 10, 11]. It is a robust, readily available modality in most hospitals. CT scanners are often located in close proximity to the trauma or emergency rooms. With the introduction of 16-slice scanners iso-volumetric imaging was possible, enabling 3D and multi-axial imaging. Sixteen-slice scanner examinations are either fast (larger collimations >1 mm) or have a high z-axis resolution (for collimations from 0.5 to 0.75 mm). The 64-slice scanners combine both fast imaging and high z-axis resolution. Dual energy and/or 256–320-slice scanners do not significantly increase z-axis resolution but are faster than 64-slice scanners. Imaging of the whole aorta (48 cm) may take 20 s (16-slice CT with small collimation), 10 s (16-slice CT with large collimation), 6 s (for 64-slice CT) or well below 3 s (for 256–320-slice or dual CT).

CT is unsurpassed in its ability to rapidly detect aortic pathology, sometimes already in non-contrasted scans (e.g., aneurysm rupture or intramural haematoma). However, non-contrast scans are not always necessary for diagnosis; multiphasic scans multiply the total radiation burden. CT angiography is usually reserved for true arterial phase CT. All aortic pathologies or associated pathologies are easily depicted in both the arterial and the delayed (venous) phase. Delayed phases are often included in an imaging protocol (in addition to CTA) to clearly depict organ malperfusion (in dissections) or identify additional venous or organ bleeding (in trauma). Table 2 shows imaging protocols for examination of different aortic pathologies.

**Table 2:** Potential protocols with multiphasic CTA to depict aortic pathologies (optional or standard parts of a multiphasic protocol)

Suspicion of	Non-contrast scan	CTA = Arterial phase (bolus triggered)	Delayed (venous) phase (+20–30 s after CTA)
Aortic trauma	Optional	Standard	Standard
Aneurysm/aneurysm rupture	Optional	Standard	Optional
Aortic dissection	Recommended	Standard	Standard
Intramural haematoma	Recommended	Standard	(Standard)
Penetrating aortic ulcer	Recommended	Standard	(Standard)
Aorto-enteric fistulae	Recommended	Standard	Standard
Aortitis	Optional	Standard	Standard

Fast imaging, quick image reconstruction and image transfer to the workstation and fast (3D) image processing are the main advantages of modern CT systems in emergency radiology. 3D vascular reconstruction with vascular segmentation and centreline reconstructions are often helpful in aortic measurements for stent graft planning. This is easier to perform in the arterial phase than in the venous phase.

The drawbacks of CT are the use of contrast media and ionizing radiation. Contrast media may pose a threat of worsening of renal function in patients with chronic renal insufficiency, although CTA imaging of the whole aorta is possible with as little as 40–50 ml of contrast with 64-slice systems (own observation).

Ionizing radiation poses a potential long-term risk especially in children and younger adults when CT is used to exclude pathology rather than to confirm it and excessive CT examination protocols are used. If CT is used for multiple follow-up examinations (EVAR, follow-up of aortic dissections) the radiation burden multiplies. In those instances the omissions of iodinated contrast media and radiation exposure are the clear advantages of (contrast-enhanced) ultrasound in the abdominal aorta (AA) or magnetic resonance imaging in the abdominal or thoracic aorta (AA or TA).

## Traumatic aortic injury

Traumatic aortic injury is a life-threatening consequence of major blunt thoracic trauma, with high early mortality if relevant trauma is untreated [3, 4]. From a clinical standpoint traumatic aortic injury has to be excluded after a relevant trauma, especially when associated with hypotension, fractures including those of the spine, scapula long bone or pelvis, pulmonary contusion and haematothorax. CT angiography is the method of choice for diagnosing aortic injury after major trauma [3].

**Fig. 1:**
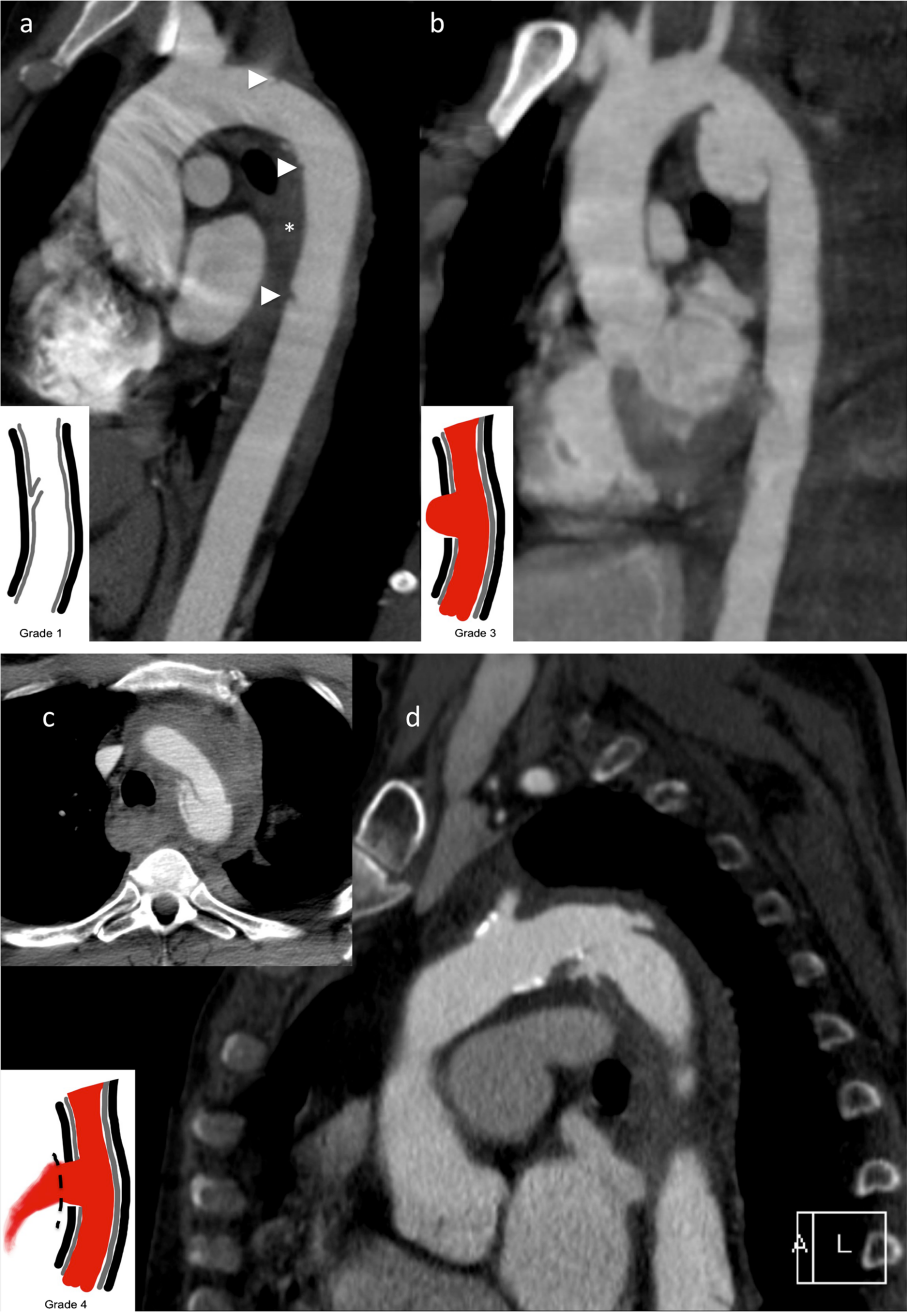
Grading of traumatic aortic injury in para-sagittal CT reformations with additional schematic presentation: (**a**) Grade 1 injury in a 38-year-old patient after a fall from 10 m. Intimal flaps are demonstrated at the level of the upper and lower curvature of the proximal descending aorta and 10 cm distal to the subclavian artery (*arrowheads*) and accompanying mediastinal haematoma. (**b**) Grade 3 injury in a 35-year-old female patient after a motor vehicle accident. A large pseudoaneurysm formation is seen in the typical position. (**c**) and (**d**) Traumatic aortic transection (grade 4) in a 79-year-old female patient after a fall from 4 m height. There is also a massive para-aortic haematoma. The patient died immediately after the CT examination

CT diagnosis relies on the confirmation of luminal irregularities (intimal flaps), outer contour irregularities (pseudoaneurysms) and periaortic haematoma, signs of contrast extravasation or frank transections of the aorta. The predilection site is the transition zone between the relatively free-moving proximal thoracic aorta (including the arch) and the fixed portion at the aortic isthmus immediately distal to the left subclavian artery. According to Azizzadeh et al. [12], and adapted by the Society for Vascular Surgery (SVS), traumatic aortic injury can be classified into four categories [12, 13]: grade I (intimal tear), grade II (intramural haematoma), grade III (pseudoaneurysm) and grade IV (rupture) (Fig. 1).

Patients with grade I can be treated conservatively [10]. Invasive treatment is currently advocated for the treatment of grades II–IV [13], with some authors recommending endovascular treatment in grade II lesions only in the presence of severe periaortic haematoma at the level of the aortic arch [14, 15]. Sometimes a staged approach for aortic repair in lower traumatic aortic injury grades (grades II and III) can be considered, treating more severe injuries (head and brain, organ pathologies) prior to the aortic injury. Nevertheless short-term follow-up to exclude progressing pseudoaneurysm or haematoma formation is recommended.

Endovascular treatment of thoracic aortic injuries is now the treatment of choice, reducing mortality and morbidity (e.g., paraplegia, renal insufficiency) compared to open surgical repair without sacrificing long-term clinical success with 5-year survival rates up to 87 % [8, 9].

**Fig. 2:**
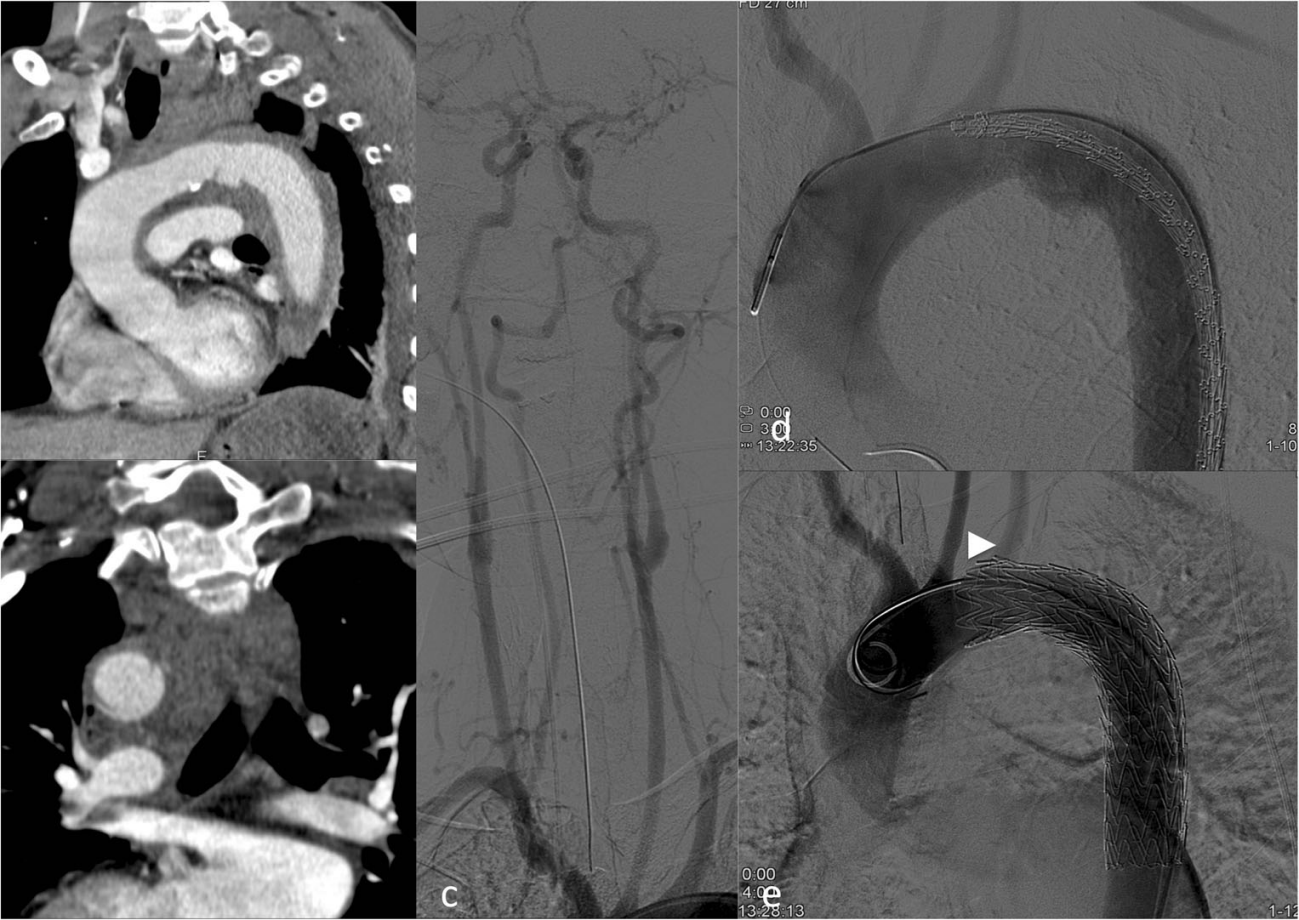
Traumatic aortic injury with stent graft implantation. A 49-year-old male patient presented with aortic trauma after a motor vehicle accident. An aortic transection with a grade 3 lesion of the proximal descending aorta (**a**) and accompanying mediastinal haematoma (**b**) is seen on a para-sagittal reformation. CTA and DSA demonstrate patency of both vertebral arteries. More pronounced pseudoaneurysm formation prior to stent graft placement and exclusion of the pseudoaneurysm after stent graft placement (**e**) over the left subclavian artery (*arrowhead*) that is still filling during control angiography

Since the predilection site for aortic injury is at the aortic isthmus in proximity to the left subclavian artery, stent graft implantation in up to 30 % of the cases will result in partial or complete coverage of the left subclavian artery [13]. Prior to coverage of the left subclavian artery patency of the right vertebral artery has to be confirmed by either initial arterial imaging up to the circle of Willis or intra-arterial angiography prior to stent graft insertion (Fig. 2). With a patent right vertebral artery of a normal calibre ischaemic complications after coverage of the left vertebral artery are very rare [16].

On centreline reconstructions it is easy to measure the length of the aortic pathology and to measure the distance from healthy to healthy vessel segments, necessary for treatment planning (= stent graft length). Correct measurement of the aortic diameter is not straightforward; the aortic diameter is dependent on the cardiac cycle (systole versus diastole) and presence or absence of hypovolaemic shock [17, 18]. This has to be taken into consideration, because stent graft oversizing of more than 15–20 % of the aortic diameter is not recommended and can lead to future complications such as retrograde dissection and stent graft collapse [17, 18].

After stent graft implantation regular CT follow-up has to be performed. How often and until when have still not been ideally determined [19]. Since the young population is usually more at risk for traumatic aortic injury the radiation burden will be substantial over time, so MRA might be a reasonable alternative to CT [20].

## Acute aortic syndrome

Initially coined by Vilacosta and Roman [21] the term “acute aortic syndrome” comprises three different but related entities:

- Aortic dissection (AD)
- Intramural haematoma (IMH)
- Penetrating atherosclerotic ulcer (PAU)

In all of these presentations, initial disruption of the medial layer of the aorta is the starting point of the vascular wall pathology. All entities share similar clinical presentations, most often sudden severe thoracic or abdominal pain, as the initial presentation. Sometimes ruptured aortic aneurysms are included under acute aortic syndrome; in this article ruptured aortic aneurysms are included as a separate chapter.

## Aortic dissection

**Fig. 3:**
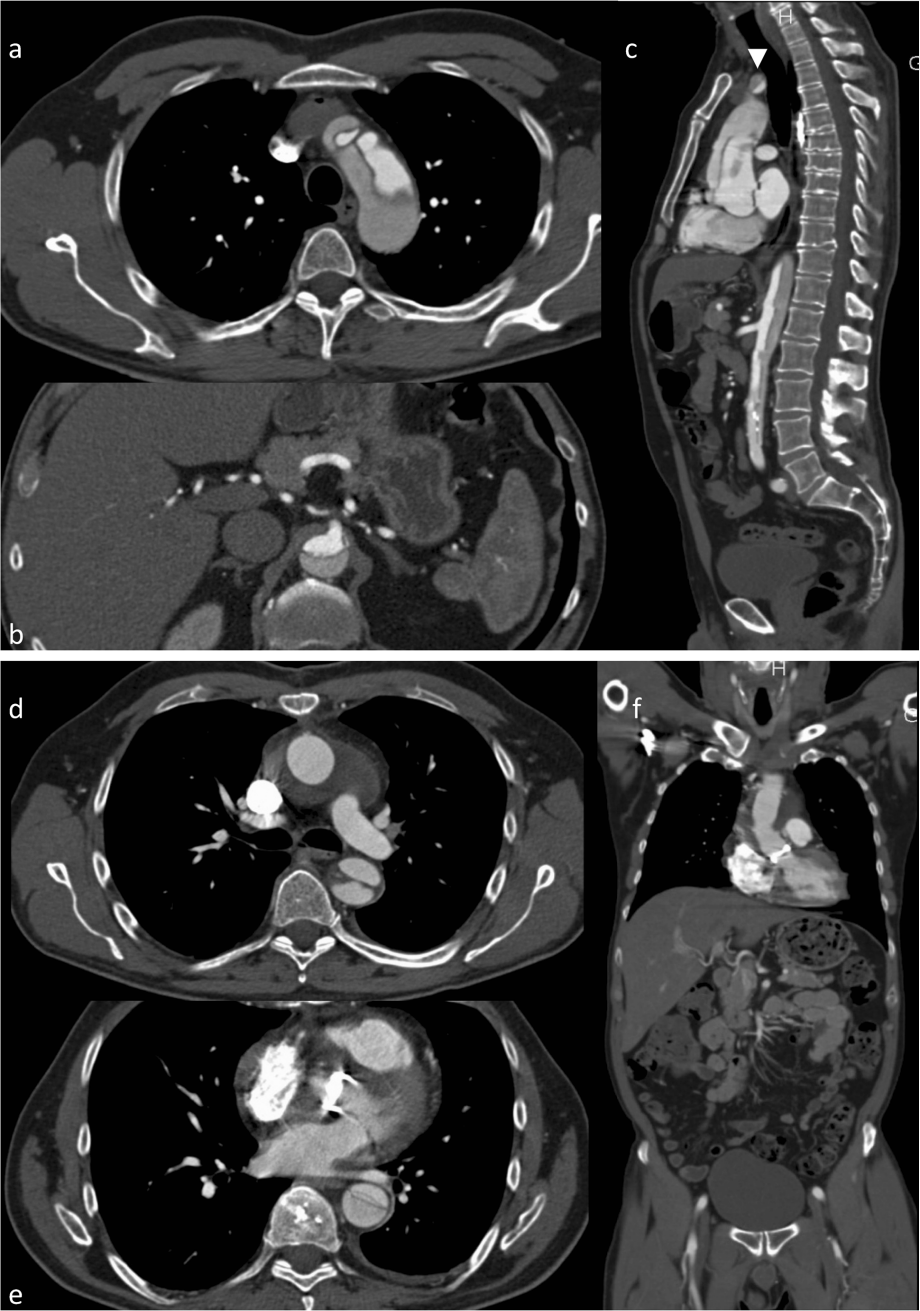

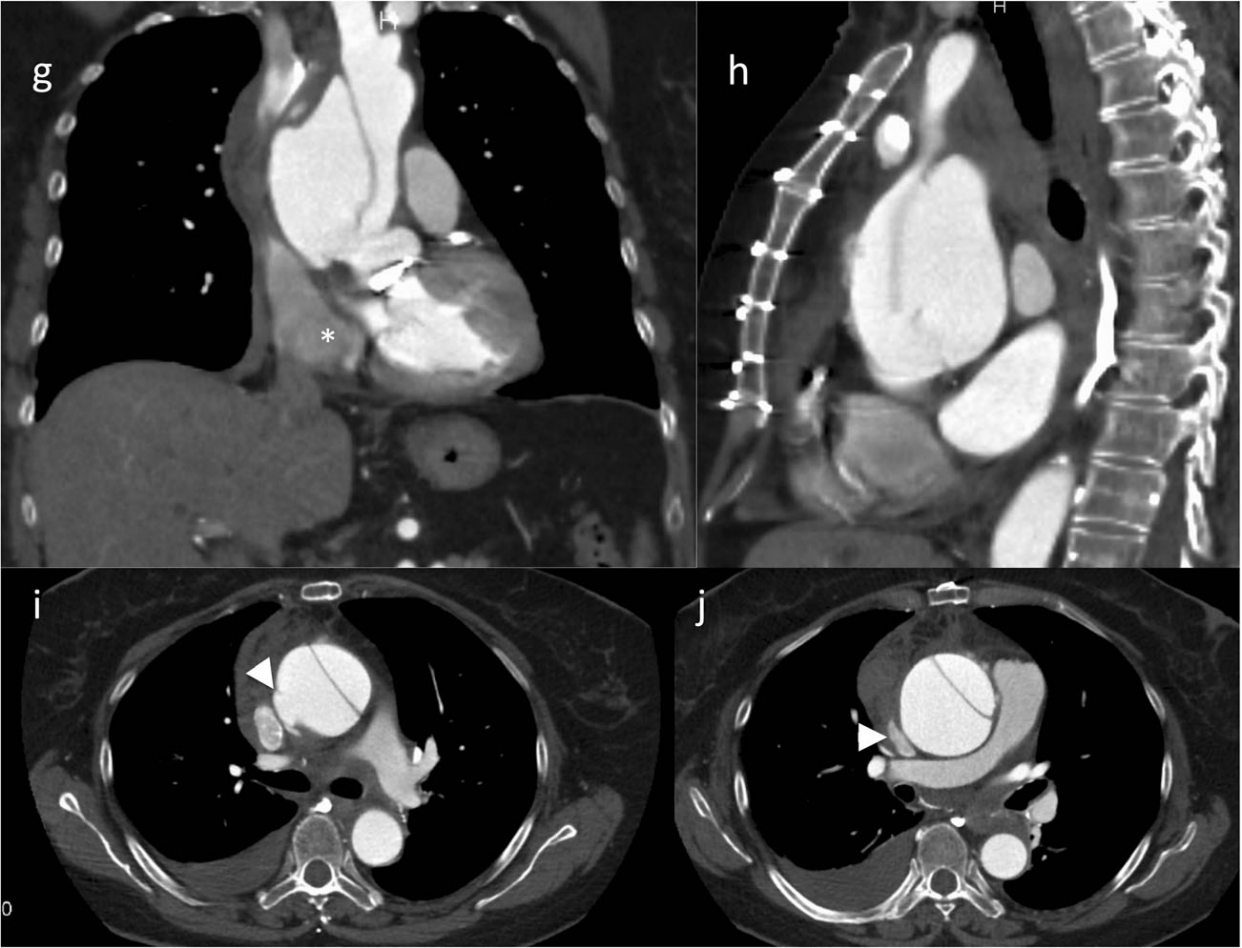
Type A dissection. A 56-year-old patient presented with chest pain in the emergency department. CTA demonstrated a type A dissection. The tear starts at the level of the sinotubular junction and extends into the supraaortic arteries (*arrowhead*) and the abdominal aorta. The false lumen opacifies to a lesser extent (and later) than the true lumen (**a**–**c**). The patient was treated with a modified Bentall procedure (composite graft replacement of the aortic valve, aortic root and ascending aorta, with re-implantation of the coronary arteries) (**c**–**e**). Ruptured type A dissection in a 71-year-old female patient (after implantation of a mechanical aortic valve). She presented with acute chest pain. CTA demonstrated a type A dissection restricted to the ascending aorta starting at the sinotubular junction [coronal (**g**) and sagittal (**h**) CT reformations]. The axial CT scans (**I**–**j**) demonstrate extravasation (*arrowhead*) and haematopericardium (*asterisk*)

**Fig. 4:**
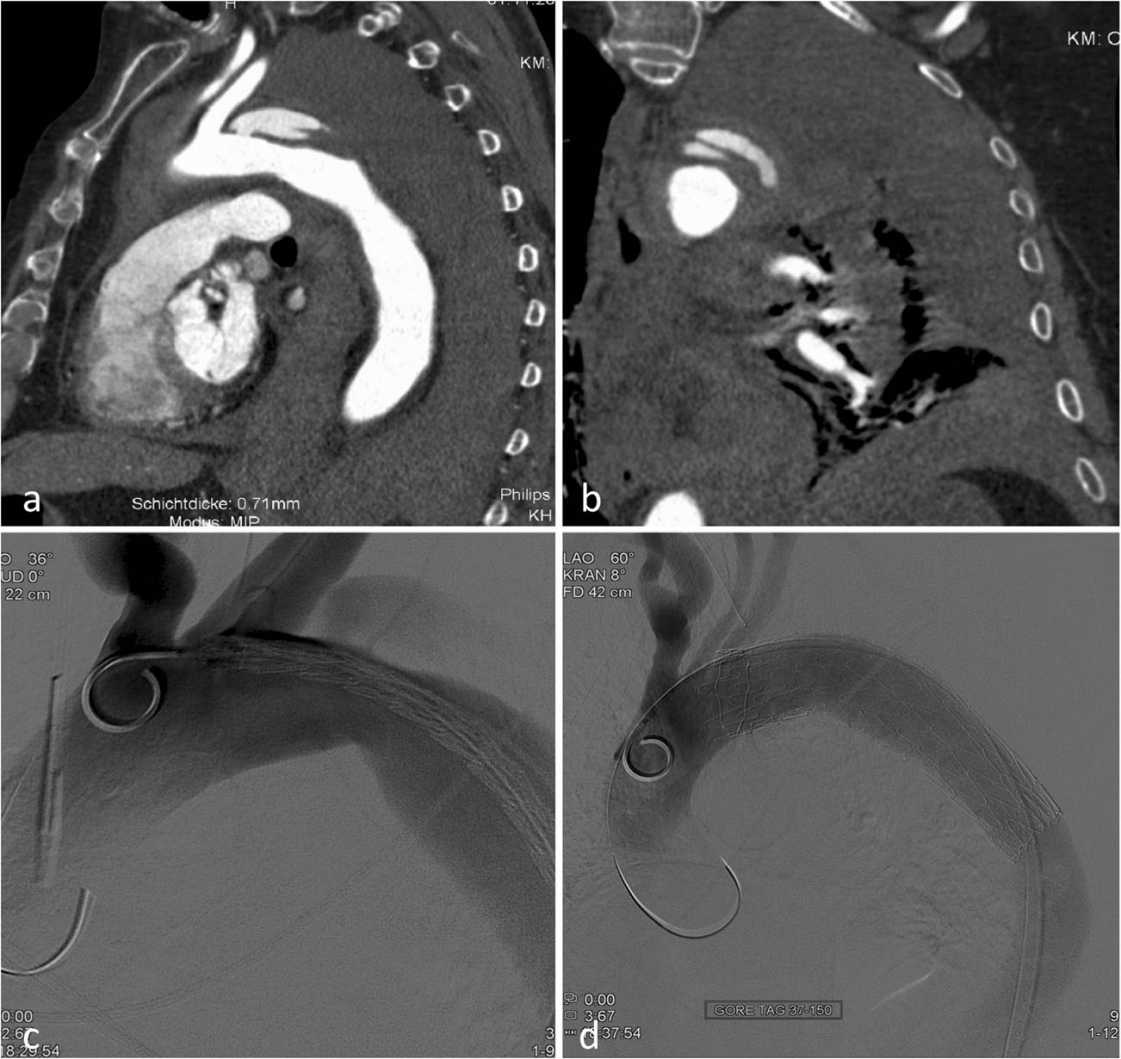
Ruptured type B dissection with stent graft implantation. An 83-year-old patient with ruptured type B dissection in para-sagittal reformations demonstrating intimal tear (**a**) and haematothorax (**a**, **b**). Stent graft implantation (**c**) (partially covering the left subclavian artery) and exclusion of the dissection after expansion of the stent graft (**d**)

**Fig. 5:**
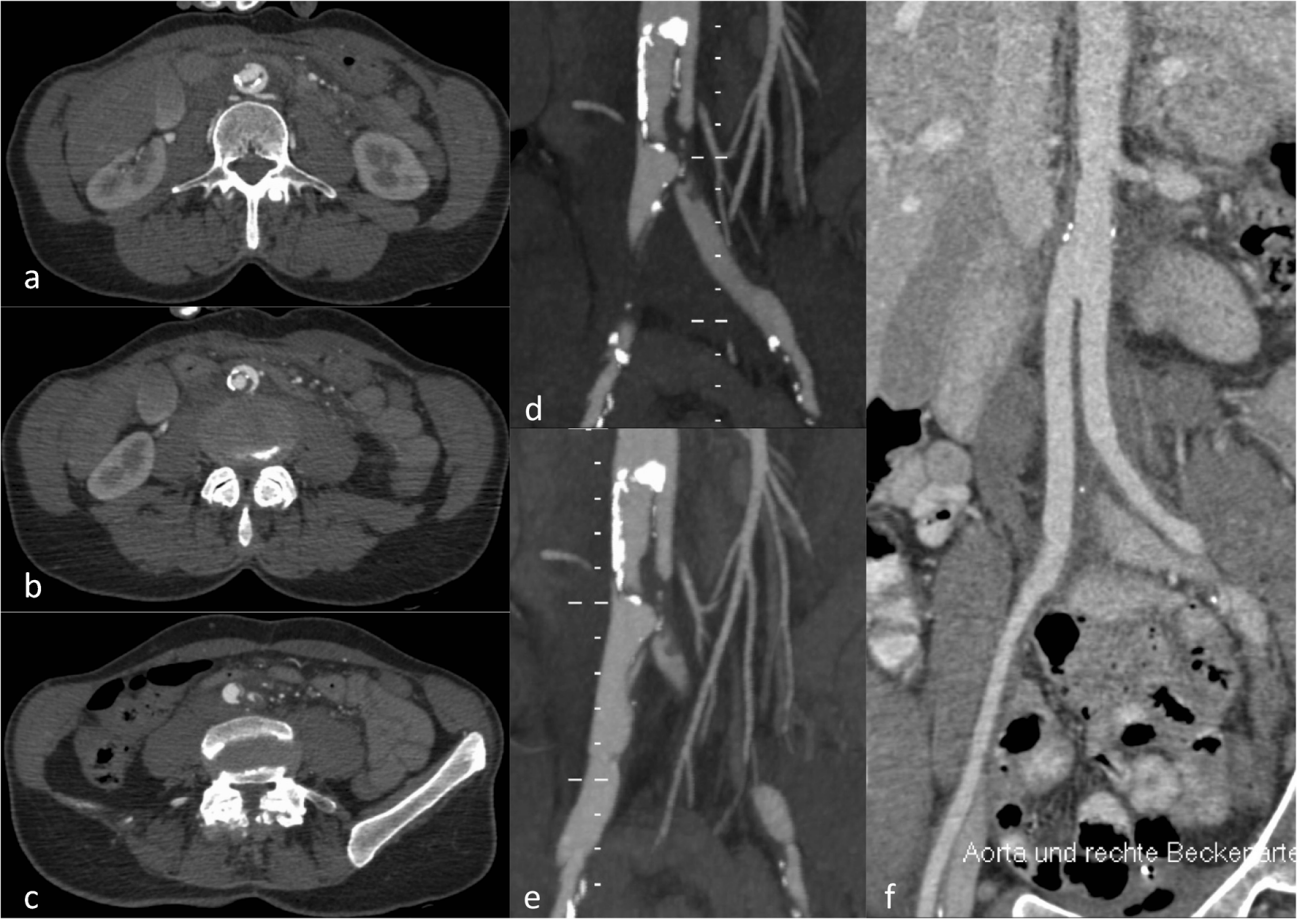
Traumatic type B dissection in the infrarenal abdominal aorta in a 55-year-old female patient who fell from 3 m height. The patient presented with abdominal pain and acute bilateral limb ischaemia caused by the dissection reaching into the common iliac artery on both sides (**a**-**e**). She was treated by a surgical approach with implantation of a bifurcated prosthesis (**f**)

An entrance tear from the lumen to the media allows blood flow into the vascular wall and therefore creates a false lumen, usually with additional communication tears between the old “true” and the new “false” lumen. The false lumen has no regular vessel wall, thus leading to aortic diameter enlargement, (pseudo) aneurysm formation and potential rupture during short- and long-term follow-up. Classification of aortic dissection is based on the location and extension of the dissection (Stanford and DeBakey classifications [3, 4]) predominantly based on involvement of the ascending versus descending versus the total aorta. The Stanford classification system grades dissections as type A dissection (in all dissections involving the ascending aorta regardless of the site of origin, surgical treatment is recommended [3, 4]; Fig. 3) or type B dissections (exclusive involvement of the descending aorta; Figs. 4 and 5).

The intimomedial (= dissection) flap separating the true from the false lumen presents either as a spiralling flap or a complete circumferential dissection. In aortic dissection it is necessary to distinguish between a true and false lumen.

The true lumen more often has a cylindrical or filiform shape. It is in clear continuity with the non-dissected proximal portion of the aorta (this is more difficult to demonstrate for type A dissections). In acute dissections it is the lumen that contains outer wall calcification. Usually the true lumen starts opacifying earlier in the arterial phase [22]. The false lumen has a characteristic “beak” appearance; this is an acute angle between the dissection flap and the outer wall of the false lumen. The false lumen starts opacifying later than the true lumen [22]. The use of retrospective gating (more radiation dose) or prospective gating (step and shoot with an option for dose-saving protocols) makes imaging of the entrance tear and re-entries (additional communications between the true and false lumen) easier than ungated CT [6].

**Fig. 6:**
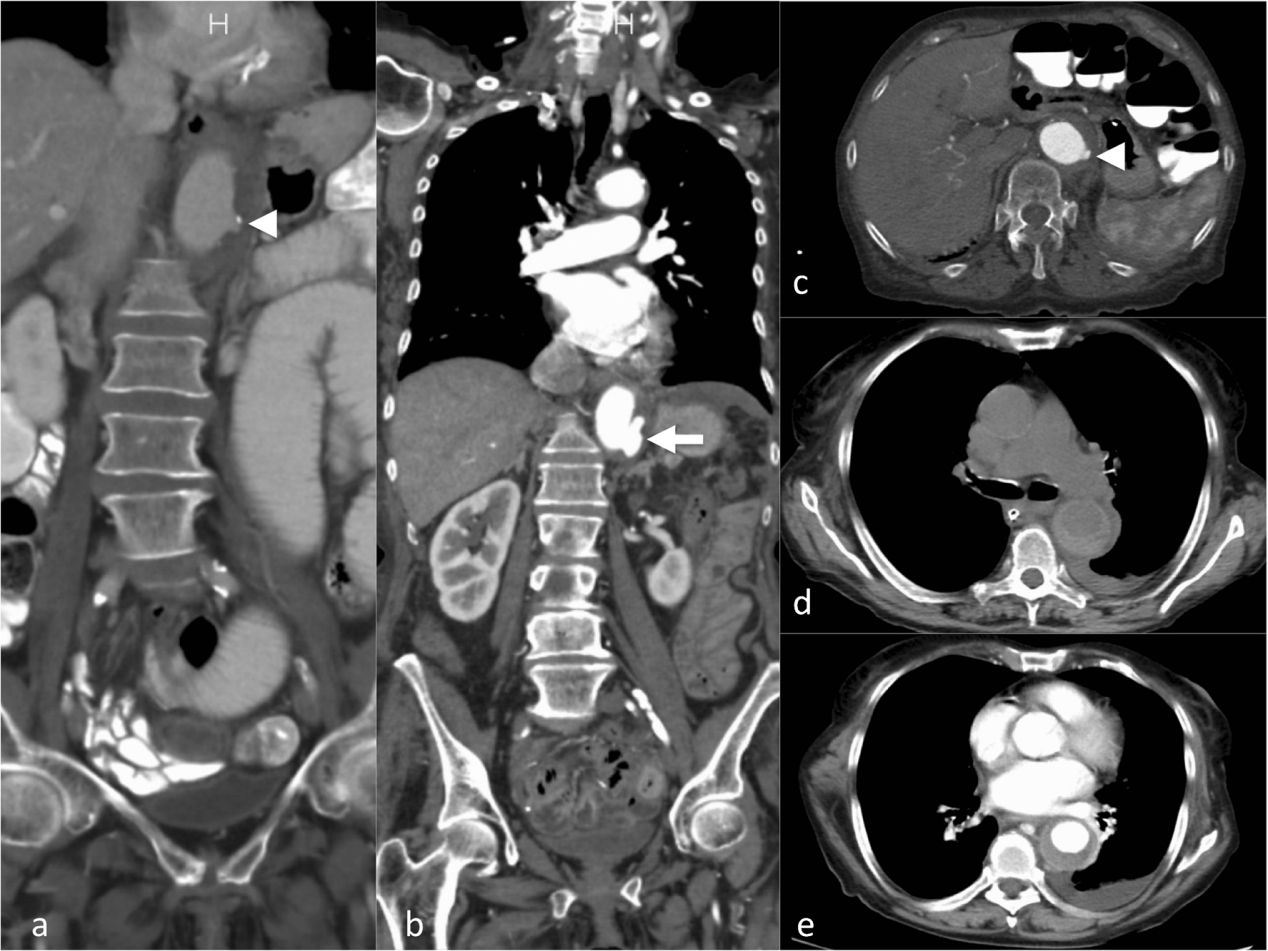
Intramural haematoma in a 79-year-old female patient. The patient presented with thoracic pain. Initial CTA with coronal reformations (**a**) and axial section (**c**) CTA demonstrated a small ulcer-like configuration (*arrowhead*) in the intramural haematoma that ascended up to the left subclavian artery (**a**). One-week follow-up CTA demonstrated enlargement of the ulcer-like configuration (*arrow*) (**b**). Native CT demonstrates the hyperdense intramural haematoma (**d**) and contrast-enhanced CT the smooth circumferential configuration of the intramural haematoma (**e**)
- **C**linical complications of the dissection (complicated versus uncomplicated dissection). A complicated type B dissection is either categorised by refractory hypertension (and pain) and/or malperfusion syndrome (in 10 % spinal, iliac and visceral arteries may be involved, clinical signs are often difficult to detect, and CT is necessary for early diagnosis) and/or haemodynamic instability (<90 mmHg systolic or at shock) and/or peri-aortic haematoma and haemorrhagic pleural effusion (with an increase on two subsequent CTs) and/or rupture or signs of impending rupture. Stent graft implantation or open surgery is usually reserved for symptomatic or complicated dissections [5] (Figs. 4 and 5). For uncomplicated dissections usually a conservative treatment approach is used. Follow-up imaging is necessary to rule out a diameter increase, pseudoaneurysm formation or progression of the dissection.
- **T**hrombus (thrombosis) within the aortic false lumen. Complete false lumen thrombosis is the key to long-term clinical success and long-term survival.

Recently a new and more complex mnemonic-based classification system has been introduced by Dake et al. [23]; the word **DISSECT** takes several clinical and imaging key factors of aortic dissection into account and therefore helps in treatment planning (medical versus endovascular versus open surgical repair):

- **D**uration of disease (acute for symptoms <14 days, subacute = 14 days to 3 months and chronic for >3 months). A marked curvature of a mobile dissection flap is more characteristic of acute dissections, whereas a rather flat and fixed appearance of an immobile and thickened flap (fibrosis leading to reduced flap mobility) is more characteristic of a chronic dissection.
- **I**ntimal tear location (also takes retrograde dissections into consideration, for example a type A dissection with an entrance tear in the descending aorta distal to the subclavian artery).
- **S**ize of the dissected aorta (in mm), rapid growth or a diameter exceeding 50 mm during follow-up warranting invasive treatment.
- **S**egmental **E**xtent (extent of aortic involvement from proximal to distal). For abdominal aortic dissection a surgical approach has some advantages because it is easier to manage the proximity and involvement of renal, visceral and peripheral (iliac) arteries (Fig. 6). The choice for surgery or stent grafting depends on the location and extent (thoracic only versus thoraco-abdominal or abdominal). Usually an open surgical approach in thoracic aorta dissection has higher morbidity and mortality than EVAR.

## Intramural haematoma (IMH)

The concept of IMH has changed over time [21, 24]. Initially it was defined as acute haemorrhage in the aortic wall, without apparent wall tear. This concept was updated after imaging [CTA, MRT or intravascular ultrasound (IVUS)] was able to depict wall tears causing the aortic wall haematoma [11]. The intramural haematoma formation is spontaneous and may be a consequence of trauma or a penetrating aortic ulcer (Fig. 6). It has been reported that IMH may progress to frank aortic dissection over time.

Non-contrast images are very helpful in depicting the more hyperdense intramural haematoma (compared to the hypodense lumen area) but not essential. In CTA the circumferential intramural haematoma also has a very characteristic appearance (Fig. 6). Indications for endovascular treatment of IMH are basically the same as for dissection [25], namely symptomatic presentation, diameter increase, pseudoaneurysm formation or progression into AD during follow-up. Imaging aims to detect the entrance tear and EVAR aims to close the entrance tear, with complete stent graft coverage of the IMH having the best therapeutic outcome.

## Penetrating aortic ulcer (PAU)

**Fig. 7:**
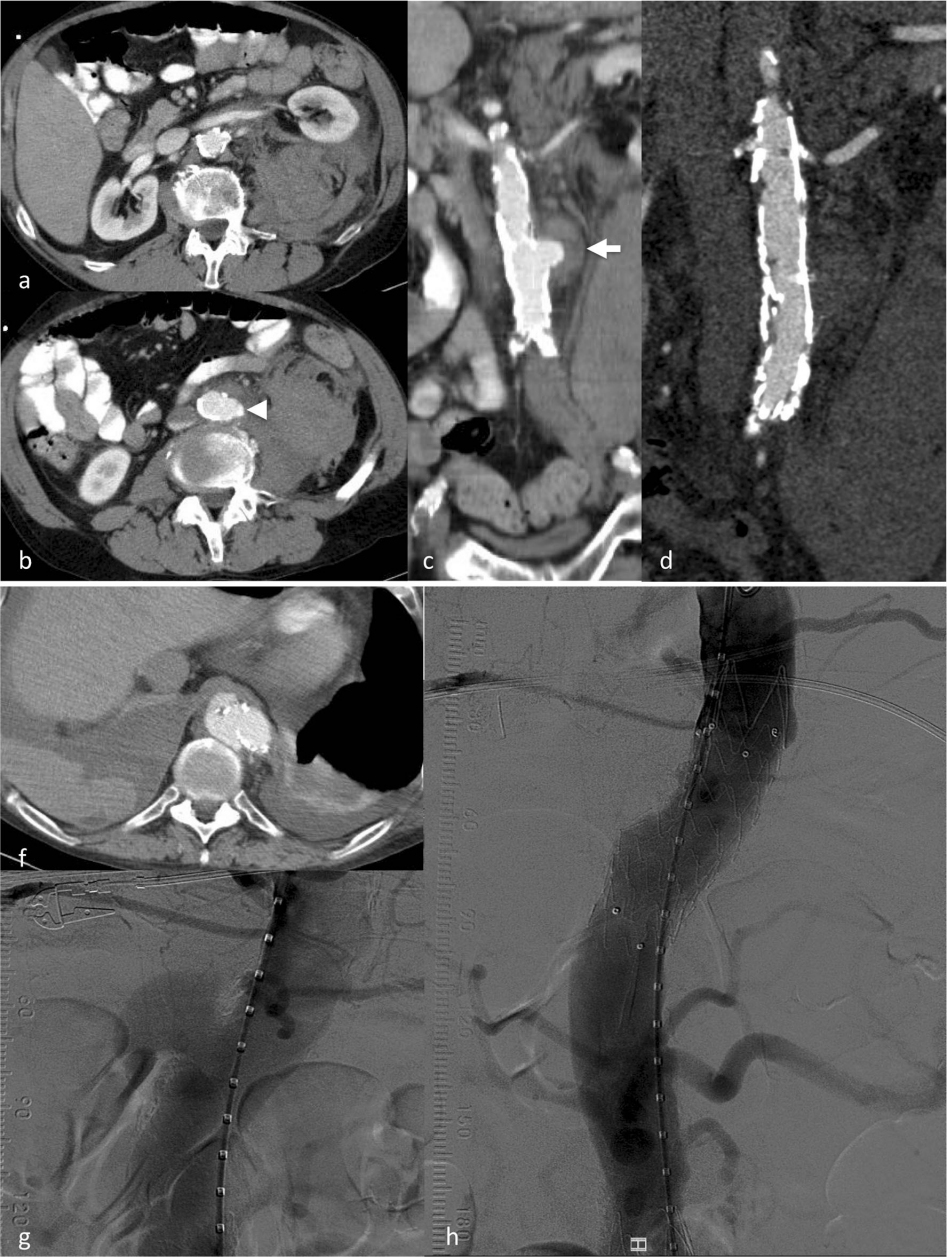
Ruptured penetrating aortic ulcer in a 57-year-old male patient. Chronic renal failure led to haemodialysis. The patient presented with hypotension and abdominal pain. CTA demonstrated a large retroperitoneal haematoma (**a**) and an aortic ulcer projecting beyond the aortic contour (*arrowhead*) (**b**). Coronal reformation demonstrated the mushroom-like appearance of the ulcer (*arrow*) (**c**). The patient was treated with implantation of a uni-iliac stent graft (**d**). Three years later he presented with hypotension and thoracic pain. CTA demonstrated a ruptured aortic ulcer in the distal descending aorta (**e**) that was again treated with implantation of a thoracic stent graft (**f**-**g**)

PAUs are most often found in patients with severe atheromatosis of the aorta or severe atherosclerosis, usually elderly patients with a multitude of co-morbidities. Atherosclerotic ulcers progress and erode the internal elastic membrane, paving the path to progression to wall haematoma [26], leading to the classic mushroom appearance (Fig. 7). The clinical (pain) and imaging presentation (growth, associated haematoma) may be the factors indicating treatment to prevent bleeding beyond the outer wall of the aorta, associated with a high risk of rupture or progression to IMH and AD (Fig. 6) [27]. Therefore, it is critical to identify PAUs as early as possible and perform regular follow-up or treatment with stent graft implantation over the penetrating ulcer [25, 28] (Fig. 7). There is currently no clear cutoff for a PAU diameter (depth) or neck diameter that warrants treatment, in one publication a depth of > 20 mm or a neck > 10 mm was associated with higher complication rates [26].

## Ruptured aortic aneurysm

Diagnosis of aneurysms depends on measurement of the vascular diameter. The normal aortic diameter is dependent on location and age. For the ascending aortic diameter, the upper limit of the normal aorta is 35 mm at the age of 20; in an 80-year-old patient the upper limit is close to 45 mm [4]. In the abdominal aorta, aneurysms are defined by a diameter >30 mm, regardless of the age of the patient. Aneurysms occur in isolation in the thoracic or abdominal aorta or can present as thoraco-abdominal aortic aneurysms. The size of the aneurysm defines the rupture risk with a clear correlation between aneurysm diameter and the risk of rupture. For AAA the annual rupture risk is 1–11 % for aneurysms between 50 and 59 mm, 10–22 % for aneurysms sized from 60 to 69 mm and peak with rupture rates of 30–33 % for aneurysms larger than 70 mm [7].

**Fig. 8:**
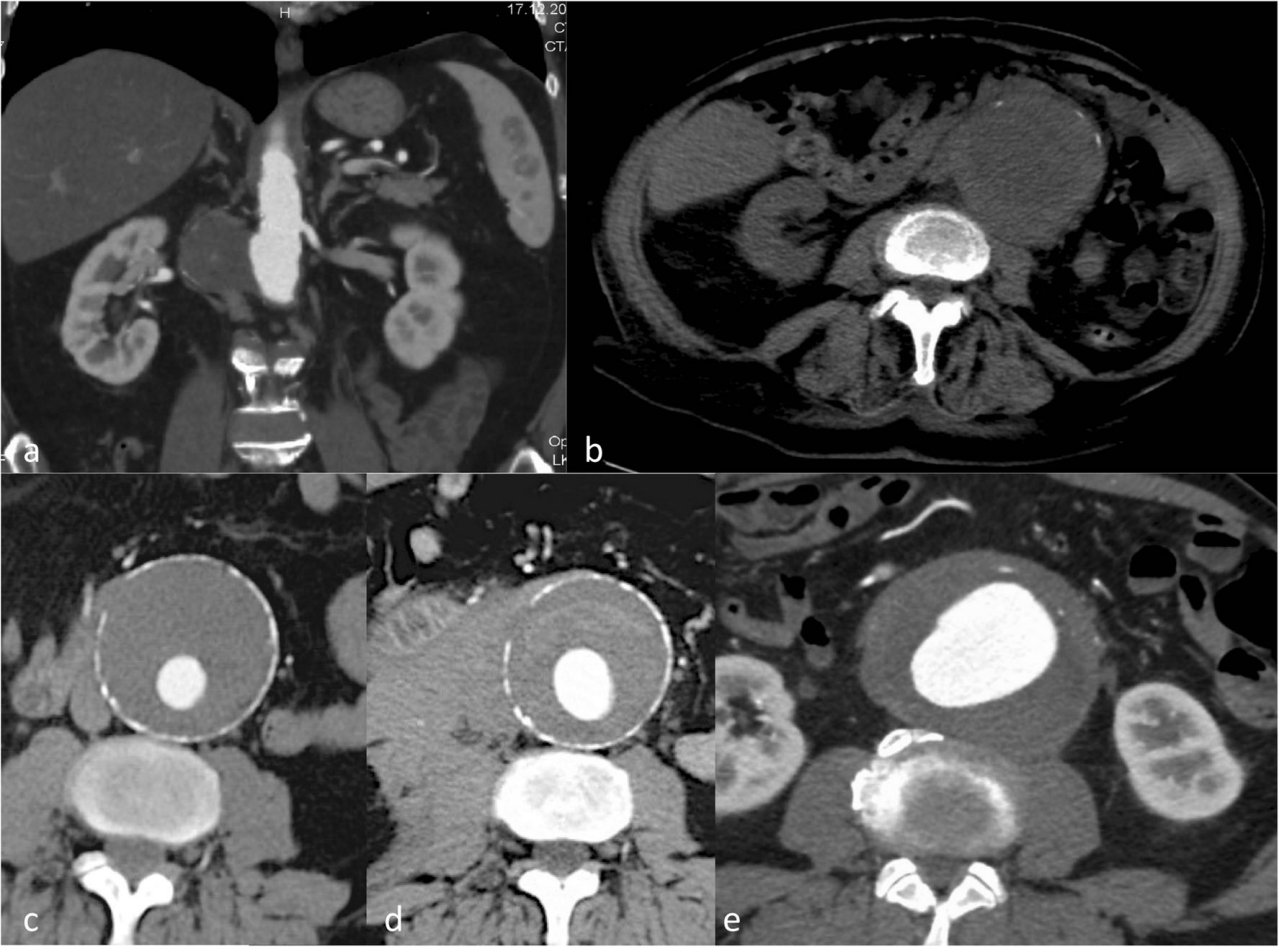
Signs of impending rupture. Focal “blebbing” (*arrow*) of the aortic wall (**a**, **c**). “Crescent sign” with hyperdense thrombus (**b**). Draped aorta signs (**b**, **e**) with flattening of the posterior aspect of the abdominal aorta. Focal “discontinuation” of the aortic contour (here with discontinuation of the wall calcification and blebbing) (**c**) in a 6-cm abdominal aortic aneurysm. The patient refused further treatment. He presented with hypotension 3 months later. CTA demonstrated a ruptured aneurysm (**d**)

There are definitive signs of impending rupture (an aneurysm size increase especially with a rapid enlargement rate, focal wall discontinuity, hyper-attenuating crescent sign, thrombus fissuration, draped aorta sign or periaortic stranding); unfortunately these signs are often not highly specific [29, 30] (Fig. 8) but detection of these findings advocates urgent treatment.

Imaging signs of completed rupture are contrast extravasation, retroperitoneal or intraperitoneal haematoma (Fig. 10), aorto-enteric or aorto-caval fistulae. AAA rupture can present as a contained rupture or free rupture. Patients with contained rupture should be managed with permissive hypotension to prevent a free rupture and keep the patient stable until treatment [31]. A free rupture can occur into the peritoneal cavity or retroperitoneal rupture with tissue tamponade reducing blood loss.

Clinical management consists of screening [especially in males (previous smokers) over 65 years], with yearly ultrasound surveillance for sizes of 30–45 mm, referral to vascular centres with CTA for aneurysms >45 mm and shorter follow-up times. Treatment is advocated when the rupture risk exceeds the risk of treatment. Currently, invasive treatment is recommended for infrarenal aortic aneurysm sizes >55 mm or upon a diameter progression of >0.5 cm/year [7]. In AAA, EVAR needs a suitable anatomy with infrarenal neck length >10 mm and neck diameters <32 mm, an appropriate diameter of access vessels (usually >6 mm) and absence of severe kinking in access vessels or in the infrarenal neck (<60°) for most of the available stent graft types. In the ascending aorta including the aortic arch, descending aorta and thoraco-abdominal aneurysms, the cutoff for treatment is a diameter of more than 6 cm [4, 7].

Symptomatic AAA is difficult to identify, detect and manage. The presenting symptoms are after non-specific abdominal pain, back pain or embolic events. When an AAA is demonstrated on imaging (regardless of size) and all other causes of abdominal pain or back pain have been ruled out (nephrolithiasis, diverticulitis, disk disease, etc.), this AAA can be considered symptomatic. Symptomatic aneurysms are thought to have a higher rupture risk than asymptomatic aneurysms; urgent surgical or endovascular repair is advocated.

**Fig. 9:**
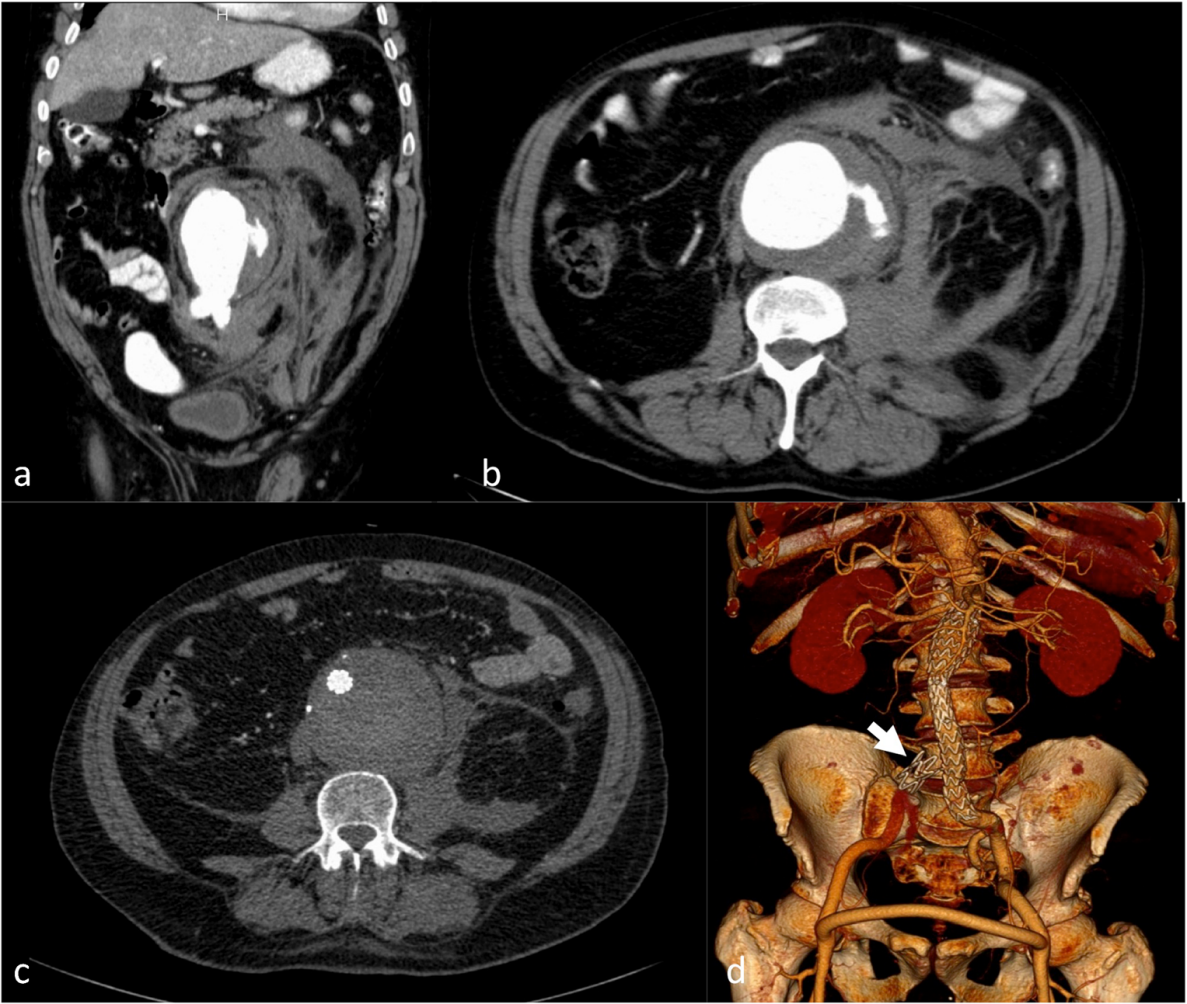
Ruptured abdominal aortic aneurysm with stent graft treatment. The 72-year-old male patient presented with left-sided abdominal pain and hypotension. CTA demonstrated a ruptured infrarenal aortic aneurysm on coronal and axial sections (**a**–**b**). The patient was treated with aorto-uni-iliac stent graft implantation (**d**) to the left, a crossover bypass and placement of an occluder (*arrow*) in the right common iliac artery. Follow-up CTA (**c**) demonstrated complete exclusion of the large aneurysm

The role of EVAR for the treatment of ruptured AAA (Fig. 9) is not completely clear. Approximately 50 % of all patients with ruptured infrarenal aortic aneurysms are suitable for EVAR. EVAR was considered to have short- and long-time survival advantages over open surgery, but this was not confirmed in a large randomised trial [32].

## Mycotic aneurysms–infected aneurysms

**Fig. 10:**
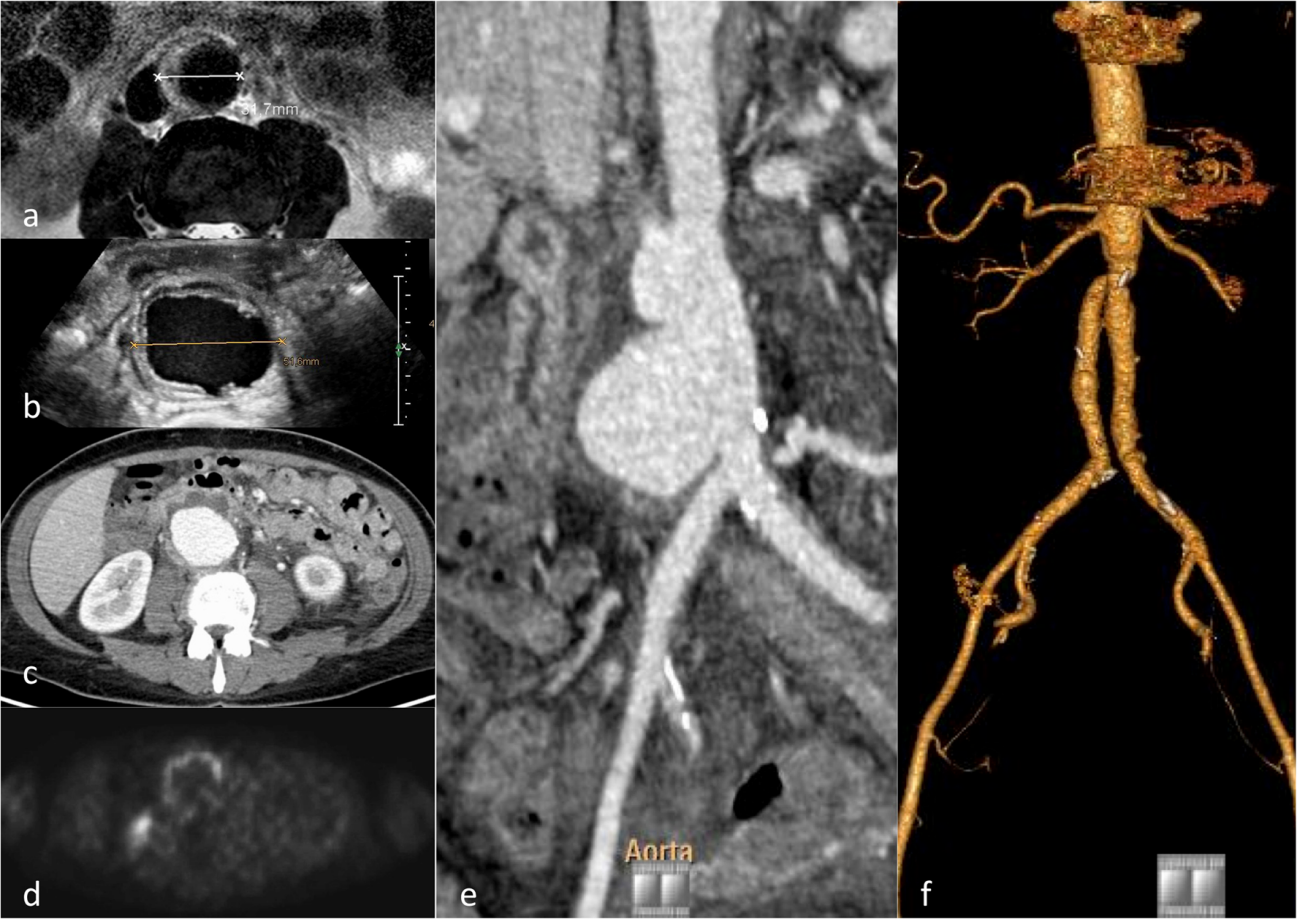
Mycotic abdominal aortic aneurysm. The 51-year-old female patient presented with abdominal/lumbar discomfort and sub-febrile temperatures. A lumbar MRI demonstrated no spondylodiscitis and a small abdominal aortic aneurysm with 35 mm diameter. One month later the abdominal aortic diameter had grown to 52 mm (**b–c**) and PET-CT (**c**–**e**) demonstrated wall thickening and PET uptake in the aortic wall and an atypical eccentric aneurysm presentation in coronal reformations (**e**). This was the only spot of infectious arthritis. Endocarditis of the mitral valve was demonstrated to be the focus. Additionally infectious arthritis of the left AC joint was treated by resection. Surgical reconstruction with deep vein (**f**) was performed; surgery confirmed a contained rupture and an aorto-duodenal fistulae

Infection is a rare cause of aortic aneurysms. Currently the term “infected aneurysm” or “infectious aortitis” is preferred over the term “mycotic aneurysm” (originally used in 1885 by Sir William Osler) since the majority of infectious agents are non-fungal (*Staphylococcus aureus*, Salmonella, Pneumococcus and *Escherichia coli* being the most common gram-positive and -negative pathogens). Mycotic aneurysms are rare (1–2 % of all aneurysms) but life threatening, usually detected when they become symptomatic. Infected aneurysms have an atypical morphological presentation, as saccular aneurysms, eccentric aneurysms or pseudoaneurysms, but they may also present as grotesque fusiform aneurysms (Fig. 10). Rapid enlargement and (PET-positive) aneurysm wall thickening and/or blurred soft-tissue structure with contrast uptake and/or abscess formation or retentions accompanying the aorta are key findings in imaging and diagnosis of infected aneurysms. Mortality after open surgery or EVAR is considerably higher than for the treatment of conventional aortic aneurysms [33, 34]. Since EVAR does not address the underlying pathology (local infection) it sometimes scores as a bridging therapy to open surgical repair; only few cases have been described where EVAR alone with long-term antibiotic treatment was a curative treatment. Only surgical treatment (resection) ± extra-anatomic reconstruction is considered a definitive treatment but with relatively high morbidity and mortality.

## Aorto-enteric fistulae (AEF)

Aorto-enteric or aorto-duodenal fistulae are rare but often fatal late complications of open repair (after graft implantation) or aneurysm infection [35, 36]. The primary diagnosis of AEF remains difficult; the clinical presentation is often with massive gastrointestinal bleeding, starting with herald bleeds and leading to severe bleeding episodes with exsanguination.

**Fig. 11:**
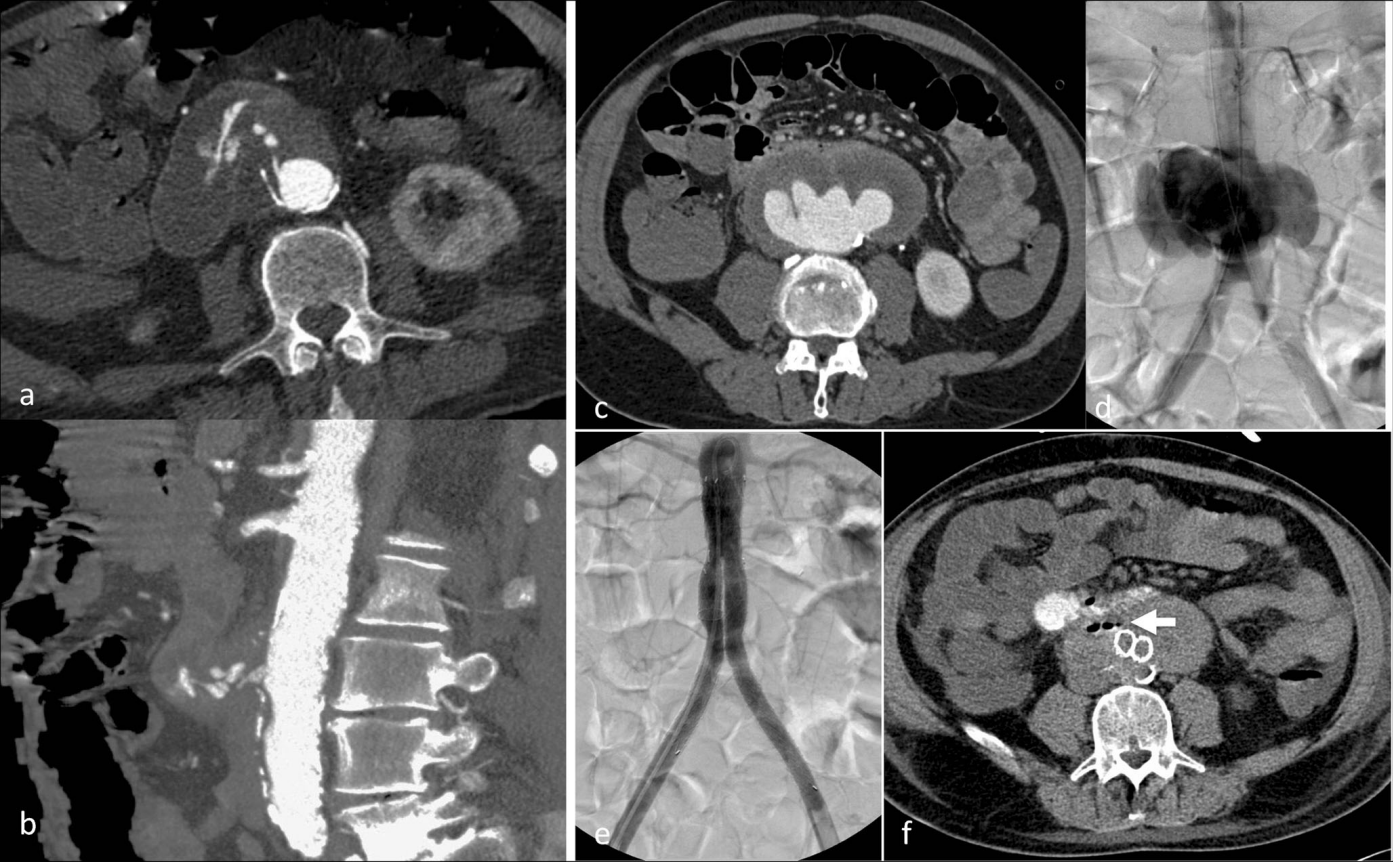
Aorto-duodenal fistulae in a 75-year-old male patient presenting with melena and haematemesis. He had an aortic repair with a surgical tube graft 10 years ago. CTA (axial and coronal reformations) demonstrates contrast extravasation of the infrarenal aorta directly into the adjacent duodenum (**a**–**b**). The patient died immediately after the CT scan before stent graft implantation could be performed. In a 63-year-old male patient with severe haematemesis ultrasound detected an aortic aneurysm. He had previous aortic repair (15 years ago) and had also been suffering from intermittent haematemesis for 3 months. CTA (**c**) and DSA (**d**) demonstrated an aneurysm of the distal anastomosis in close proximity to the duodenum. A bifurcated stent graft was implanted (**e**). The aneurysm was completely excluded. A follow-up CT on the next day (after oral contrast) nicely demonstrated the aorto-duodenal fistulae (*arrow*) (**f**)

Just like in infected aneurysms computed tomography is the diagnostic imaging of choice in the acute phase. CT imaging findings suggestive of aortic infections (air/gas in the thrombus) are confirmed by PET-CT (fluorodeoxyglucose positron emission computed tomography) to be of infectious origin. Demonstration of contrast extravasation from the aorta to the duodenum is diagnostic of an acute bleeding aorto-enteric fistula. Therapy consists of an urgent individual interdisciplinary approach, potentially combining EVAR for bridging and open surgery as the definitive treatment approach (Fig. 11).

## Iatrogenic aortic injury

**Fig. 12:**
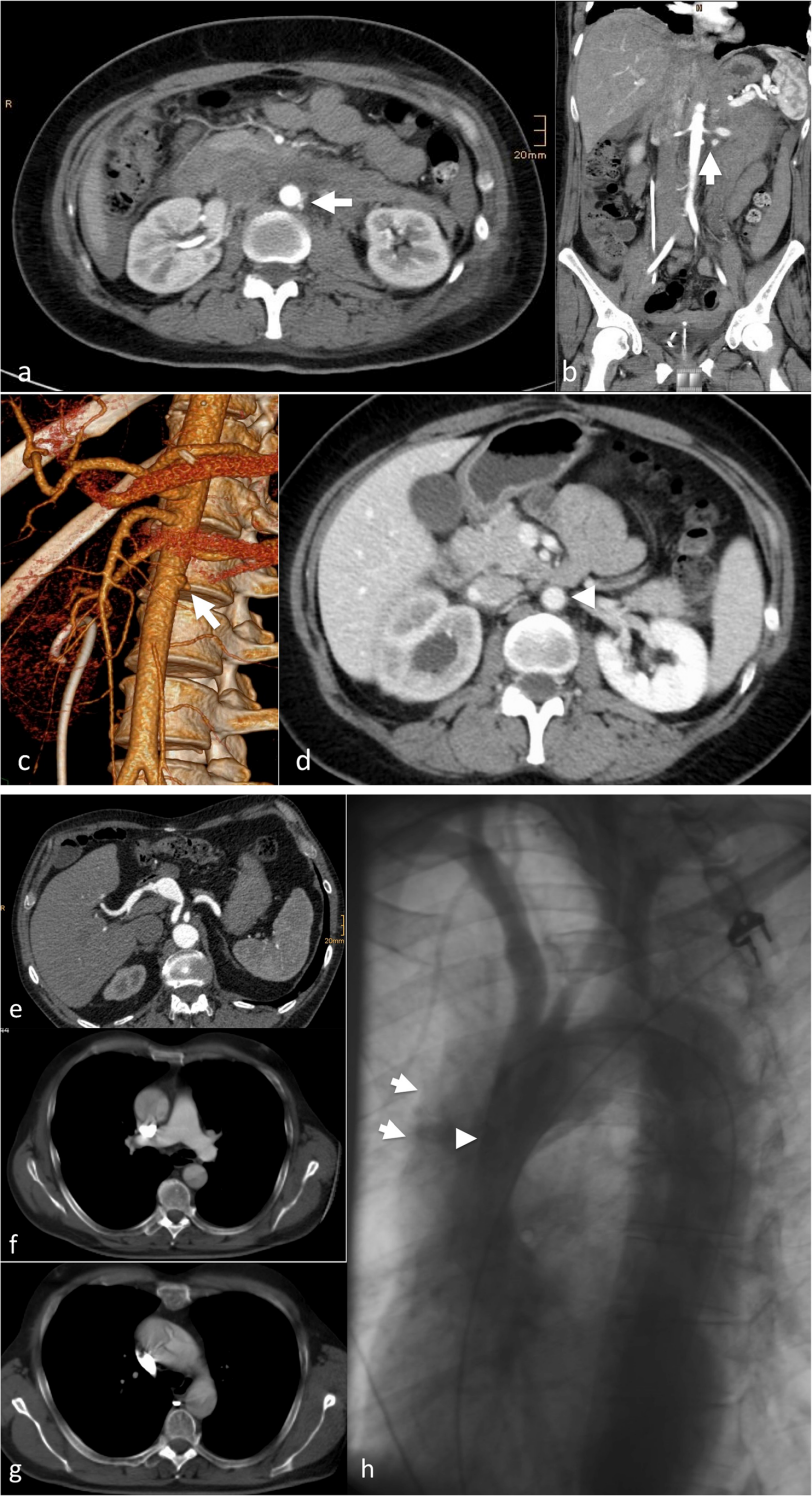
Iatrogenic aortic injury. A 36-year-old female patient underwent laparoscopic removal of an ovarian cyst; 6 h later she presented with hypotension and severe abdominal pain. CTA [(**a**) axial section, arrowhead; (**b**) coronal reformation; (**c**) volume-rendered image, *arrow*] demonstrated a left lateral laceration of the aorta, confirmed to be a trocar injury during vascular surgical reconstruction. A 6-month follow-up CT demonstrates a rather smooth aortic contour at the level of the original injury (**d** *arrowhead*). A 66-year-old patient presented with an aneurysm of the coeliac trunk/common hepatic artery (**e**). During an endovascular approach for stent graft implantation, when the Simmons type II catheter was configured in the aortic arch, the patient reported severe thoracic pain; DSA (**f**) and CTA [(**g**–**h**) on a single slice CT scanner] confirmed the suspicion of a type A aortic dissection with a true lumen (*arrowhead*) and false lumen (*arrows*) on unsubtracted angiographic images

There are various mechanisms for iatrogenic aortic injury. On one hand it can be caused by direct external injury from surgical procedures, causing direct bleeding or (delayed) pseudoaneurysm. If detected intraoperatively, it can be treated by a direct surgical approach. Delayed bleeding or pseudoaneurysm formation is usually detected by CTA or by surgical revision (Fig. 12a–d). On the other hand it may be caused by an endovascular approach. The larger the catheters or introduction materials (sheaths, etc.) are, the higher the number of access (= iliac) vessel complications (rupture, dissection, occlusion), which are by far the most common complications [37]. With the introduction of stent grafting, endovascular aortic valve implantations and aortic balloon pumps, the numbers of direct aortic injuries have increased, albeit not by much. Dissections are the most common type of injuries, but they are not exclusively caused by large-calibre devices; they can also be caused by small catheters (Fig. 12e–h).

Retrograde aortic (type A) dissections have been reported as complications of stent graft implantation for the treatment of type B dissections or descending aortic aneurysms in the literature [38] and may also be considered an iatrogenic injury.

## Summary

Aortic emergencies are relatively rare, but they have to be detected very quickly. CTA is the imaging method of choice and helps to decide whether elective, urgent or emergent treatment is necessary. EVAR and surgical repair are the treatment approaches of choice.
